# Sociodemographic and clinical predictors in patients with borderline personality disorder: A 12-month follow-up study

**DOI:** 10.1017/S1092852926101084

**Published:** 2026-07-14

**Authors:** Andrea Aguglia, Gabriele Sapia, Francesco Stefanini, Michela Gagliardi, Noemi Redaelli, Annapaola Mazza, Benedetta Conio, Alessandra Costanza, Mario Amore, Andrea Amerio, Gianluca Serafini

**Affiliations:** 1 Department of Neuroscience, Rehabilitation, Ophthalmology, Genetics, Maternal and Child Health, Section of Psychiatry, University of Genoa, Genoa, Italy; 2 AOM - IRCCS Ospedale Policlinico san Martino, Genoa, Italy; 3 ASL 2 Savona (SV), Italy; 4 Department of Psychiatry, Faculty of Medicine, Geneva University (UNIGE), 1205 Geneva, Switzerland; 5 Department of Psychiatry, Faculty of Biomedical Sciences, University of Italian Switzerland (USI), 6900 Lugano, Switzerland

**Keywords:** Borderline personality disorder, hopelessness, alexithymia, substance abuse, rehospitalization

## Abstract

**Objective:**

The care of patients with borderline personality disorder (BPD) places a significant burden on emergency inpatient mental health services. The aim of this study was to investigate predisposing risk factors for early rehospitalization over 12 months.

**Methods:**

A total of 117 inpatients with BPD were included. The Beck Hopelessness Scale, Adolescent/Adult Sensory Profile, Coping Orientation to Problems Experienced (COPE), and Toronto Alexithymia Scale (TAS-20) were administered.

**Results:**

Forty-seven percent of the sample was rehospitalized. Divorced marital status, current substance abuse, a higher score on the TAS-20 externally oriented thinking score and on the COPE Alcohol and drug disengagement subscales, and a lower score on the COPE. Use of social–emotional support subscale were significantly associated with rehospitalization. Therefore, alcohol and substance misuse, impaired social support, and alexithymia were the most relevant predictors for rehospitalization.

**Conclusions:**

Maladaptive coping, depressive symptoms, and history of high service utilization and multiple high-lethality suicide attempts were also found to be more common in readmitted subjects. Further research could help to better define potential different therapeutic interventions.

## Introduction

Borderline Personality Disorder (BPD) is a severe psychiatric disorder described as a condition characterized by a pervasive pattern of instability in the sense of self, problematic interpersonal relationships and emotional dysregulation accompanied by fluctuations in cognitive state, including transitory psychotic and dissociative experiences. Recurrent feelings of emptiness and relational stressors can find expression in acute crises characterized by nonsuicidal self-harm, suicide attempt, or substance intoxication, which often lead to emergency services and hospitalization.

The diagnostic criteria of BPD have remained stable from the Diagnostic and Statistical Manual of Mental Disorders, third edition (DSM-III), published in 1980 to the DSM-5-TR,^1^ which retains a diagnostic threshold of at least five criteria and allows for 256 potential combinations of symptoms, suggesting a heterogeneous diagnostic category.^2^ A recent study proposed a categorization of the broad constellation of symptoms that characterize the clinical presentation of BPD, distinguishing three different symptom-based subtypes: nonlabile type—characterized by the absence of emotional instability and dissociative symptoms; dissociative/paranoid type—defined by low fear of abandonment, low identity disturbance, and high levels of dissociation and paranoid ideation; interpersonally unstable type—marked by frequent efforts to avoid abandonment, aggressiveness, and a pattern of alternating idealization and devaluation in interpersonal relationships.^3^ The etiology of BPD is not completely understood, and recent scientific findings suggest a multifactorial pathogenesis involving genetic predisposition, neurobiological alterations, and a history of childhood trauma.^4^ Linehan theorized a biosocial model of pathogenesis in which biological vulnerabilities interact with an invalidating environment, amplifying emotional reactivity and intermittently reinforcing and perpetuating problem behaviors, which represent maladaptive attempts to regulate crises.^5^ BPD has an estimated prevalence ranging from 1% to 3.9% in the general population, approximately 10% among outpatients,^6^ and 15–28% among psychiatric inpatients.^7^ Suicide rates are 52 times higher than those in the general population,^8^ about 50–90% of patients suffering from BPD have made at least one suicide attempt (SA), and a 3–10% die by suicide. Furthermore, a history of nonsuicidal self-injury (NSSI) has been reported in about 70 to 80% of adult patients suffering from BPD, and is considered a risk factor for later suicidal behavior.^9^ ^,^^10^ The stratification of suicide risk, reason for further psychiatric hospitalization, includes the presence of comorbid mood disorders, substance use disorders, history of prior suicide attempts, presence of early and severe traumatic experiences, and coexisting antisocial personality traits, all of which may be associated with greater severity of borderline psychopathology.^11^ ^–^^13^ The psychosocial functioning and quality of life of these patients are severely impaired, as indicated by frequent presentations to emergency services and a high risk of rehospitalization with similar prevalence rates of other severe psychiatric disorders, making BPD management and treatment one of the topics of priority interest in national healthcare guidelines.

To the best of our knowledge, no studies to date have explored the factors associated with the risk of rehospitalization among individuals with a primary diagnosis of BPD in Italy. The aim of this study was to identify sociodemographic, clinical, and psychopathological dimensions as potential risk factors associated with early rehospitalization in a cohort of Italian patients over a 12-month follow-up period.

## Materials and methods

### Sample

For this study, one hundred seventeen inpatients with a primary diagnosis of BPD, according to DSM 5^14^ criteria, were consecutively recruited at the Psychiatry section of the AOM - IRCCS Ospedale Policlinico San Martino —Department of Neuroscience, Rehabilitation, Ophthalmology, Genetics, Maternal and Child Health, University of Genoa, Italy, between January 2019 and November 2023. Furthermore, the diagnosis was made through the administration of the SCID-5 PD form by expert psychiatrists without any involvement in this study.^15^

After enrollment at baseline (T0), each patient was followed for 12 months to monitor the first rehospitalization after discharge. Time was measured in months from discharge to the rehospitalization or the end of the 12-month follow up. After discharge, all patients were referred to a local Mental Health Service (CSM) in ASL 3 Genovese for personalized outpatient psychiatric care and clinical follow-up.

Inclusion criteria for enrollment in the study were (a) primary diagnosis of BPD, (b) current age ≥18 years old, and (c) written informed consent signed by the patient. Exclusion criteria were: (a) lack of clinical documentation; (b) patient receiving outpatient care outside the catchment area of ASL 3 Genovese; (c) refusal to provide a written informed consent; (d) psychiatric disorders considered clinically predominant over BPD at the time of hospitalization, including schizophrenia spectrum disorders, bipolar disorder, severe major depressive episodes, or substance use disorders requiring primary clinical management. Anxiety disorders and secondary psychiatric comorbidities were not considered exclusion criteria when BPD represented the principal diagnosis and primary reason for hospitalization; and (e) comorbid acute medical conditions.

An in-depth explanation of the study objectives and procedures with the opportunity to ask questions were provided to all participants, explaining the aim of the current study and the potential clinical impact in terms of personalized treatment to prevent rehospitalization. The study was conducted according to the Declaration of Helsinki guidelines^16^ and was approved by the local Ethical Review Board.

### Assessment

A semistructured interview, used in previous studies,^17^ ^,^^18^ was administered by senior residents in psychiatry to collect sociodemographic and clinical data. The information was integrated with the electronic medical records of our hospital’s digital system. The following information was recorded: gender, age, marital status, educational level, psychiatric family history, duration of hospitalization, number of voluntary admissions, lifetime prevalence of involuntary psychiatric hospitalizations, current and lifetime alcohol, substance and medication abuse, current suicidal ideation, current, lifetime, and number of SA. Lethality of SA was defined as “the acquired ability to enact lethal self-injury”^19^. High-lethality suicide attempt (HLSA) and low-lethality suicide attempt (LLSA) were identified, according to our previous studies.^20^ ^,^^21^

Subsequently, different psychometric tools were administered to each patient to investigate specific clinical dimensions. The evaluation scales were performed by expert psychiatrists with years of experience with the following psychometric tools, once the acute psychopathological condition was considered more stable, as follows:

1. the Adolescent/Adult Sensory Profile (AASP): it is a 60-item self-administered questionnaire used to assess sensory processing patterns. Participants are asked to report the frequency with which they respond to everyday sensory experiences by selecting one of five options: *almost never*, *seldom*, *occasionally*, *frequently*, or *almost always.* Based on their responses, individuals are categorized into one of four sensory processing quadrants: low registration, sensation seeking, sensory sensitivity and sensation avoiding.^22^
2. the Beck Hopelessness Scale (BHS): a 20-item true-false scale designed to quantify hopelessness. Each item is scored as either 0 or 1, and the total “hopelessness score” is calculated as the sum of all item scores, ranging from 0 to 20.^23^
3. the Coping Orientation to Problems Experienced Inventory (COPE): a psychometric scale designed to measure the frequency of different coping strategies individuals employ when facing stressful situations. This self-report instrument consists of 60 items, divided into 15 subscales (positive reinterpretation and growth, mental disengagement, focus on and venting of emotions, use of instrumental social support, active coping, denial, religious coping, humor, behavioral disengagement, restraint, use of emotional social support, substance use, acceptance, suppression of competing activities, planning). These subscales assess three overarching dimensions of coping: problem-focused coping, emotion-focused coping and potentially maladaptive strategies.^24^
4. the Toronto Alexithymia Scale (TAS-20): a 20-items scale designed to assess alexithymia. The instrument includes three subscales: difficulty identifying feelings (DIF), difficulty describing feelings (DDF) and externally oriented thinking (EOT). Each item is rated on a Likert-type scale, and the total score ranges from 20 to 100.^25^

### Statistical analysis

The Statistical Package for the Social Sciences for Windows 25.0 was used to perform statistical analyses (IBM Corp., Armonk, NY, USA). Statistical significance was set at *p* < 0.05.

Continuous and categorical variables were presented as means and standard deviations or frequencies and percentages, respectively. The total sample was divided into two subgroups, according to the criteria of rehospitalization after the index event within 12 months. The comparisons between categorical variables were evaluated performing chi-squared tests, while Student’s t-test for independent samples was used to evaluate differences between continuous variables. Furthermore, time-to-event analysis was conducted with a Kaplan–Meier estimation of the survival function with a monthly interval plot to illustrate the probability of staying out of hospital. A Cox logistic regression analysis was performed to adjust the impact of sociodemographic and clinical associated with the subgroups and test predictive value. The regression included all variables, which were found to be statistically significant between the two subgroups at t-test or chi-squared test.

## Results

### Cohort characteristics

A total of 117 patients with a primary diagnosis of BPD were included in this study, with a prevalence of females (71.8%) and an average current age at enrollment of 33.56 ± 13.73 years. A total of 55 subjects (47%) experienced rehospitalization during the 12-month follow-up. The divorced status was found more associated with the probability of being rehospitalized (40.0% vs.17.7%; *p* = .045), confirmed by the Cox regression (*p* = .014, *HR* 2.025). All sociodemographic characteristics are summarized in Table 1.

**Table 1.** Sociodemographic Characteristics of the Total Sample and According to Rehospitalization Within 12 Months Table 1. Long description.

	Total sample (*N* = 117)	Rehospitalized (*N* = 55)	Non-rehospitalized (*N* = 62)	*t/χ* ^2^	*p*
Gender (female)	84 (71.8)	37 (67.3)	47 (75.8)	1.048	.306
Current age in years	33.56 ± 13.73	34.60 ± 13.55	32.65 ± 13.94	−.767	.445
Marital status					
Single	53 (45.3)	22 (40.0)	31 (50.0)	8.045	**.045**
Married	25 (21.4)	8 (14.5)	17 (27.4)		
Divorced	33 (28.2)	22 (40.0)	11 (17.7)		
Widowed	6 (5.1)	3 (5.5)	3 (4.8)		
Educational level in years	11.80 ± 2.95	11.45 ± 2.99	12.11 ± 2.90	1.209	.229

The statistical significance is signed by bold value.

From the comparison of clinical characteristics investigated, several statistical differences were found. Specifically, a longer duration of hospitalization in days (12.18 ± 7.83 vs.9.03 ± 7.39, *p* = .027), more than lifetime five admissions in a psychiatric ward (47.3% vs.24.2%, *p* = .027), a higher prevalence of a lifetime HLSA (32.7% vs.12.9%, *p* = .036), and higher number of suicide attempts (2.05 ± 2.67 vs.1.06 ± 1.72, *p* = .017) were associated with the rehospitalization within 12-month follow-up. Furthermore, the presence of current alcohol (56.4 vs.33.9%, *p* = .015) and substance (60.0% vs.32.3%, *p* = .003) abuse was related to the probability of being rehospitalized. Only the current substance abuse was found as a predictor in the Cox regression analysis (*p* = .031, *HR* 1.868). All clinical characteristics are displayed in Table 2.

**Table 2.** Clinical Characteristics of the Total Sample and According to Rehospitalization Within 12 Months Table 2. Long description.

	Total sample (*N* = 117)	Rehospitalized (*N* = 55)	Non-rehospitalized (*N* = 62)	*t/χ* ^2^	*p*
Family history for psychiatric disorder	62 (53.0)	28 (50.9)	34 (54.8)	.181	.671
Duration of hospitalization in days	10.51 ± 7.73	12.18 ± 7.83	9.03 ± 7.39	−2.238	**.027**
Involuntary admissions	32 (27.4)	12 (21.8)	20 (32.3)	1.599	.206
Voluntary admissions					
No	16 (13.7)	5 (9.1)	11 (17.7)	7.208	**.027**
< 5 hospitalizations	60 (51.3)	24 (43.6)	36 (58.1)		
≥ 5 hospitalizations	41 (35.0)	26 (47.3)	15 (24.2)		
Current suicide ideation	58 (49.6)	30 (54.5)	28 (45.2)	1.027	.311
Current suicide attempt					
Low-lethality	19 (16.2)	9 (16.4)	10 (16.1)	.052	.975
High-lethality	7 (6.0)	3 (5.5)	4 (6.5)		
Lifetime suicide attempts					
Low-lethality	33 (28.2)	13 (23.6)	20 (32.3)	6.660	**.036**
High-lethality	26 (22.2)	18 (32.7)	8 (12.9)		
Number of suicide attempts	1.53 ± 2.26	2.05 ± 2.67	1.06 ± 1.72	−2.412	**.017**
Alcohol abuse lifetime	70 (59.8)	38 (69.1)	32 (51.6)	3.704	.054
Alcohol abuse current	52 (44.4)	31 (56.4)	21 (33.9)	5.972	**.015**
Substance abuse lifetime	63 (53.8)	34 (61.8)	29 (46.8)	2.654	.103
Substance abuse current	53 (45.3)	33 (60.0)	20 (32.3)	9.052	**.003**
Medication abuse lifetime	48 (41.0)	24 (43.6)	24 (38.7)	.292	.589
Medication abuse current	30 (25.6)	18 (32.7)	12 (19.4)	2.734	.098

The statistical significance is signed by bold value.

### Psychometric tools

The comparison of the two subgroups in terms of the psychometric tools showed a significant correlation between rehospitalization and higher BHS total score (11.80 ± 5.75 vs.9.63 ± 5.68, *p* = .042), TAS-20 total score (60.05 ± 14.70 vs.52.29 ± 15.29, *p* = .006), TAS-20_DDF (16.18 ± 5.78 vs.13.82 ± 5.68, *p* = .028), and TAS-20_EOT (20.87 ± 5.57 vs.17.76 ± 4.61, *p* = .001).

Regarding coping strategies, rehospitalized patients with BPD showed lower total score on the COPE problem-focused coping, specifically on the “use of instrumental social support” subscale (9.80 ± 2.98 vs.11.02 ± 2.32, *p* = .015) and on COPE emotion-focused coping, specifically on the “use of social–emotional support” (9.40 ± 2.79 vs.11.02 ± 2.38, *p* = .001) and “positive reinterpretation and growth” (9.82 ± 2.76 vs.11.10 ± 2.19, *p* = .006) subscales. Finally, regarding COPE potentially disadaptive strategies, the “alcohol and drug disengagement” subscale reported higher total score in the rehospitalized subgroup (10.02 ± 3.20 vs.8.23 ± 2.91, *p* = .002). No statistically significant differences were found on the sensory profile scale. All findings are shown in Table 3.

**Table 3.** Clinical Dimensions Investigated of the Total Sample and According to Rehospitalization Within 12 Months Table 3. Long description.

	Total sample (*N* = 117)	Rehospitalized (*N* = 55)	Non-rehospitalized (*N* = 62)	*t/χ* ^2^	*p*
AASP—Low registration	36.03 ± 11.85	37.07 ± 12.47	35.11 ± 11.29	−.892	.374
AASP—Sensation seeking	44.40 ± 8.79	44.35 ± 9.01	44.45 ± 8.59	.065	.948
AASP—Sensory sensitivity	42.94 ± 10.50	42.33 ± 10.01	43.48 ± 10.97	.593	.554
AASP—Sensation avoiding	40.97 ± 10.89	40.80 ± 11.99	41.11 ± 9.92	.154	.878
Beck hopelessness scale	10.65 ± 5.79	11.80 ± 5.75	9.63 ± 5.68	−2.053	**.042**
Toronto alexithymia scale-20 (TAS-20)	55.94 ± 15.45	60.05 ± 14.70	52.29 ± 15.29	−2.791	**.006**
TAS-20_DDF	14.93 ± 5.83	16.18 ± 5.78	13.82 ± 5.68	−2.224	**.028**
TAS-20_DIF	21.83 ± 7.89	23.09 ± 7.46	20.71 ± 8.16	−1.641	.104
TAS-20_EOT	19.21 ± 5.29	20.87 ± 5.57	17.76 ± 4.61	−3.294	**.001**
COPE problem-focused coping					
Active coping	10.68 ± 2.66	10.71 ± 2.87	10.65 ± 2.48	−.129	.897
Planning	10.31 ± 2.96	10.25 ± 3.16	10.35 ± 2.78	.182	.856
Suppression of competing activities	10.00 ± 2.41	10.05 ± 2.64	9.95 ± 2.20	.353	.819
Restraint coping	9.23 ± 2.13	9.16 ± 2.06	9.29 ± 2.21	.320	.750
Use of instrumental social support	10.44 ± 2.71	9.80 ± 2.98	11.02 ± 2.32	2.477	**.015**
COPE_emotion-focused coping					
Use of social–emotional support	10.26 ± 2.69	9.40 ± 2.79	11.02 ± 2.38	3.385	**.001**
Positive reinterpretation and growth	10.50 ± 2.55	9.82 ± 2.76	11.10 ± 2.19	2.791	**.006**
Acceptance	9.91 ± 2.57	9.85 ± 2.77	9.95 ± 2.40	.203	.839
Humor	8.85 ± 3.36	8.60 ± 3.44	9.08 ± 3.30	.771	.442
Venting of emotions	11.39 ± 2.54	11.44 ± 2.46	11.35 ± 2.63	−.173	.863
Turning to religion	7.32 ± 3.80	7.44 ± 3.87	7.21 ± 3.77	−.321	.749
COPE_potentially disadaptive strategies					
Denial	8.74 ± 2.86	8.36 ± 2.88	9.08 ± 2.81	1.360	.176
Behavioral disengagement	9.03 ± 2.66	9.80 ± 2.98	9.80 ± 2.98	.269	.788
Alcohol and drug disengagement	9.07 ± 3.17	10.02 ± 3.20	8.23 ± 2.91	−3.173	**.002**
Mental disengagement	10.34 ± 2.42	10.16 ± 2.26	10.50 ± 2.57	.748	.456

The statistical significance is signed by bold value.

Table 4 and Figure 1 reported the findings resulted from Cox regression analysis, identifying the potential significant predictors of rehospitalization of patients with a primary diagnosis of BPD.

**Table 4.** Cox Regression to Establish Predictors of Rehospitalization Table 4. Long description.

	*p*	HR	95% CI
Marital status (divorced)	.014	2.025	1.154–3.554
Current substance abuse	.031	1.868	1.059–3.296
TAS-20_EOT	.035	1.063	1.004–1.126
COPE_Use of social–emotional support	.034	.887	.793–.991
COPE_Alcohol and drug disengagement	.026	1.103	1.012–1.204

**Figure 1.**
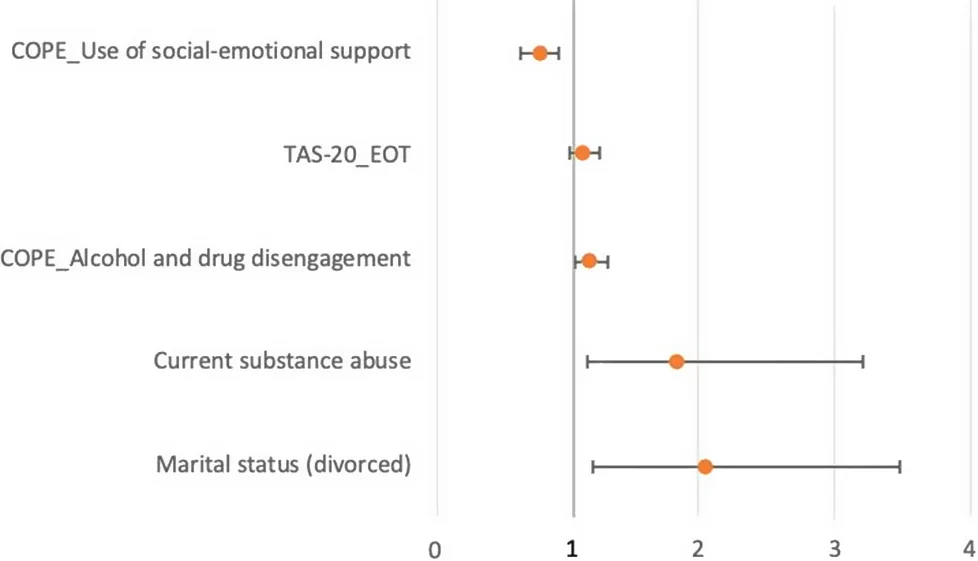
Forest plot representing hazard ratio with 95% confident interval. Figure 1. Long description.

The Kaplan plot shows the trend in rehospitalization in the 12-month follow-up (Figure 2), with steeper drops clustered in the first months after discharge and trending to a plateau around the sixth month.

**Figure 2.**
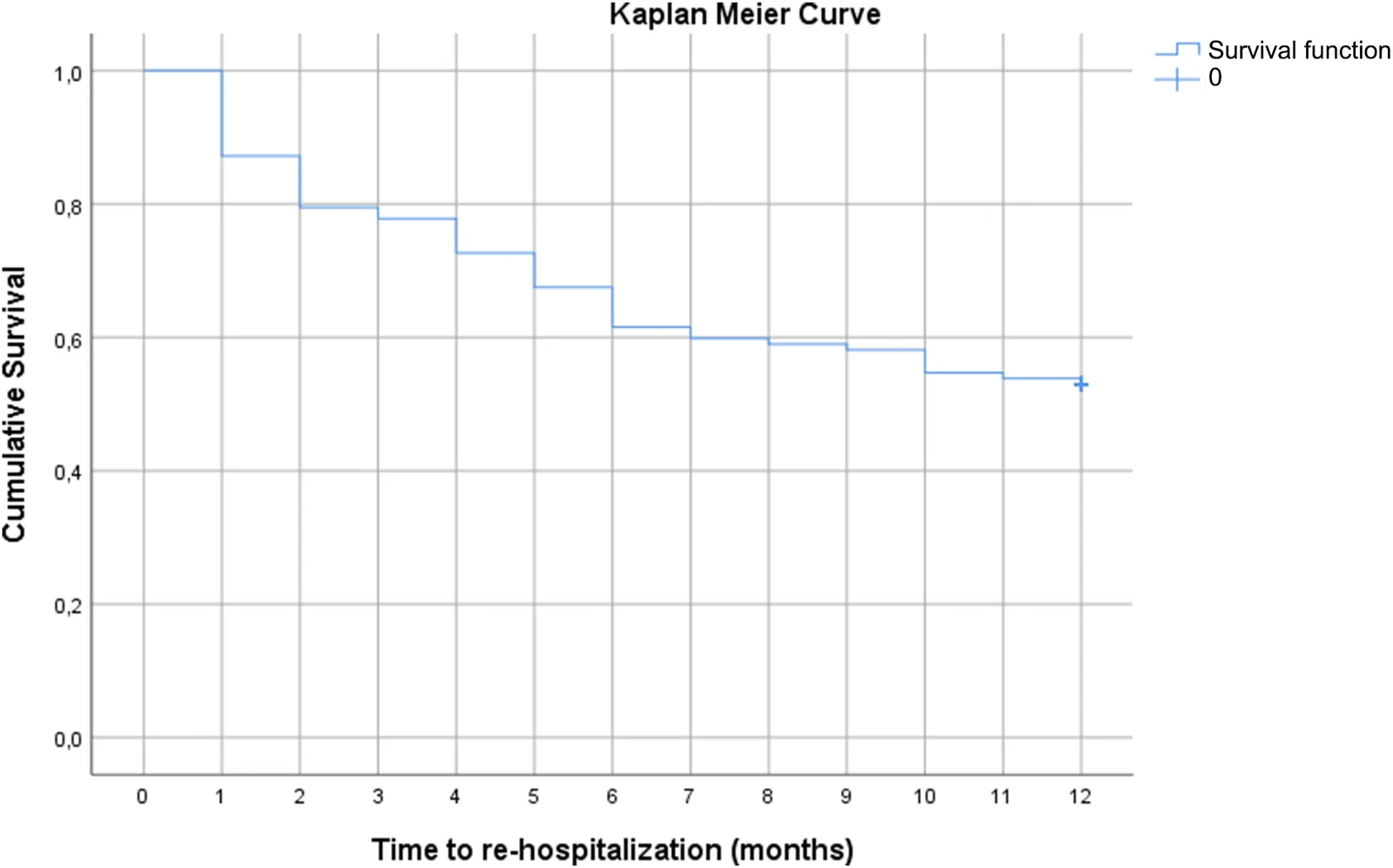
Kaplan-Meier Plot of re-hospitalization after 12 months from the index event. Figure 2. Long description.

## Discussion

The aim of this study was to investigate sociodemographic, clinical characteristics, and psychopathological dimensions as potential predisposing factors for early rehospitalization in patients with BPD during a 12-month follow-up. In the time-to-event analysis of the total sample, 47% of the total sample was rehospitalized within 12 months with a nonlinear risk trajectory concentrated in the early postdischarge window, consistent with broader psychiatric rehospitalization literature, reflecting the spike in adverse outcomes immediately after discharge from hospital (“revolving door” dynamic).^26^ ^–^^29^

Length of stay in a psychiatric ward, lifetime suicidality burden, current alcohol or substance use, higher hopelessness and alexithymia scores (particularly EOT), and lower social support coping were found more prevalent in rehospitalized patients. In the Cox regression analysis, only separated/divorced marital status, current substance abuse, alexithymia with high EOT scores and “alcohol and drug disengagement”—“use of social–emotional support” coping strategies were shown to be the most impactful clinical predictors. Survival analysis illustrated a substantial risk of early rehospitalization linked with higher illness severity indexes. Our findings support the interpretation that early rehospitalization reflects the intersection between the domain of emotional dysregulation and inadequate containment in the community, echoing Linehan’s model of innate sensitivity versus invalidating environment. Early postdischarge contact, such as a telephone check-in within 48 hours and face-to-face appointments within 7 days, together with support for alcohol/substance misuse and integrated dual-diagnosis pathways could reduce the so-called “revolving door dynamics” through personalized and specific therapeutic strategies. The implementation of intensive postdischarge interventions and structured psychotherapeutic pathways may be challenging in routine clinical settings, particularly in community mental health services with limited resources and variable access to evidence-based treatments such as dialectical behavior therapy (DBT). In this context, the identification of clinical and psychosocial predictors associated with early rehospitalization could help clinicians stratify patients according to the risk and prioritize longitudinal monitoring and follow-up interventions for subjects with greater clinical complexity. Furthermore, while some predictors identified in our study, such as substance misuse and maladaptive coping strategies, may represent potentially modifiable therapeutic targets, marital status should be considered a nonmodifiable risk factor and interpreted primarily as a marker of psychosocial vulnerability requiring closer clinical monitoring rather than a direct intervention target.

Literature investigating the relationship between marital status and risk of rehospitalization in patients with BPD is relatively limited. A recent study found divorced marital status as a predictor of a shorter time between further hospitalizations compared to married status.^30^ Another Italian study, evaluating a broader and heterogeneous psychiatric population, similarly suggested that patients who were generally categorized as unmarried tended to be at greater risk of experiencing multiple hospitalizations over 12-month observation period.^31^ Therefore, our findings could be interpreted considering the tendency of patients with BPD to experience unstable interpersonal relationships, a condition that may lead to divorce.^32^ Furthermore, limited “use of social–emotional support” coping strategy could be interpreted as either or both contributing factor and effect of said relational instability. Conversely, being married could be considered a proxy for higher psychosocial functioning and potentially lower illness severity at time of observation, and it acts as a protective factor against rehospitalization in the general psychiatric population.^27^

Although regression analysis did not confirm hopelessness feelings as reliable predictors for the increased risk of rehospitalization in our sample, hopelessness as well as lifetime suicidal behaviors (in terms of lifetime high-lethality and number of suicide attempts) were significantly reported at univariate analysis, supporting the literature evidence that hopelessness is considered a transdiagnostic vulnerability factor tied to suicidality, heavier service use, and self-medication patterns in patients with BPD. Furthermore, reduced use of social–emotional support and greater reliance on denial mechanisms were independently associated with hopelessness, offering a plausible bridge between coping strategies and subsequent crisis services utilization.^18^

In patients with BPD, alcohol and illicit substance abuse is highly prevalent.^33^ Both DSM-5-TR and International classification of diseases—11th revision^34^ included substance abuse in the diagnostic criteria. It is estimated that up to 78% of individuals with BPD present a comorbid substance use disorder.^35^ This comorbidity has been linked to greater illness severity in patients with BPD^36^ and our findings indicated that current substance use at the time of recruitment constitutes a risk factor for early rehospitalization. The use of alcohol and substances for coping strategies in patients with BPD was confirmed.^37^ ^,^^38^ To best our knowledge, there is no study exploring substance or alcohol misuse in BPD, specifically using COPE. The role of alcohol and substance misuse in amplifying risk of adverse outcomes is well known, and the undermining of inhibitory control may be especially relevant in individuals with high impulsivity and low stress tolerance.

Our study revealed that mean TAS-20 scores in the overall cohort were clustered around the “possible alexithymia” cut-off. Rehospitalized patients exhibited significantly higher mean TAS-20 total score and higher score on the TAS-20 EOT and DDF subscales. Several studies have shown that alexithymia was considered a recurrent feature in patients with BPD, and is associated with suicidal ideation and NSSI. Moreover, high TAS-20 EOT score has been correlated with use of emergency care services.^39^ ^–^^43^ A recent review highlighted that alexithymia contributes to the severity of BPD, affecting treatment compliance negatively.^44^ Individuals reporting high TAS-20 EOT score displayed a tendency of concrete cognitive style to external events rather than affect recognition and integration with a consequent deficit in cognitive–emotional processing.^45^ DDFs associated with EOT were linked to misreading of social cues, interpersonal stress reactivity, and escalation to self-injury, pathways that plausibly channel risk toward rehospitalization when emotional support is unavailable or ineffectively used.^46^ It is possible that this externally focused mode of thinking, when combined with core affective features of BPD, such as recurrent dysphoria and emotional dysregulation, may limit patients’ ability to process and integrate stressful experiences, increasing their vulnerability to psychopathological crises and recurrent hospitalizations. However, alexithymia and coping strategies were assessed during acute psychiatric hospitalization, a phase frequently characterized by severe emotional distress and crisis-related dysregulation. Therefore, although alexithymia is generally considered a relatively stable psychological construct, some dimensions—particularly EOT—may also be partially influenced by state-dependent factors related to the acute clinical condition. Similarly, maladaptive coping strategies observed during hospitalization may have been amplified by the ongoing psychopathological crisis. Future longitudinal studies, including repeated assessments during outpatient stabilization, could help better distinguish stable trait characteristics from acute state-related fluctuations. Nevertheless, this hypothesis would fit with data, supporting psychoeducational and validating approaches such as those used in DBT as relapse preventive intervention. It could help clinicians to plan and organize better the therapeutic intervention in light of the presence of maladaptive coping strategies in rehospitalized patients, with emotion- and problem-focused coping scores lower in the event group as indicated by both instrumental (ie financial help, physical resources) and emotional social support along with the positive reinterpretation and growth subscale. These results seem to fit with the DBT dialectical dilemma of active passivity versus apparent competence, which describes one origin of BPD patients’ difficulties in enacting effective help-seeking strategies.^47^ Moreover, lower “positive reinterpretation and growth” scores in rehospitalized patients could explain the tendency in patients with BPD to remain adherent to familiar patterns of dysfunctional behaviors, maintaining high levels of caregiver burden.^48^ The ability to effectively rely on emotional social support in one’s community acts as a protective factor against rehospitalization as indicated by COPE subscales, confirmed by proportional hazard analysis,^49^ describing perceived emotional support and positive reframing as indicators of lesser illness burden.

The strength of this study is the prospective design, but several limitations should be considered before interpreting our findings. First, our sample was relatively small, including only inpatients from a single psychiatric unit and considering only severe cases. In addition, patients presenting psychiatric disorders considered clinically predominant over BPD at the time of hospitalization were excluded from the study. This methodological choice was made to reduce diagnostic overlaps; however, it may have introduced a selection bias toward patients with less diagnostically complex clinical presentations. In real-world settings, BPD is frequently associated with comorbid mood and substance use disorders, which are themselves linked to greater functional impairment, increased suicidality, poorer treatment adherence, and higher service utilization. Therefore, excluding patients with those clinically predominant comorbidities at the time of hospitalization may have resulted in a cohort with a partially lower psychopathological burden but reduced clinical heterogeneity compared to typical inpatient BPD populations. Consequently, the generalizability of our findings to broader psychiatric settings may be limited, particularly in patients with high complex dual diagnosis who represent a substantial proportion of emergency and inpatient psychiatric admissions. Our study also did not account for psychological factors that could have contributed to clinical differences between patients.

## Conclusions

Overall, a high risk of early rehospitalization in our clinical sample was associated with indicators of greater illness severity, including numerous previous admissions, a higher lifetime number of SAs enacted with high-lethality methods, higher hopelessness and alexithymia scores, maladaptive coping strategies, and worsened by impaired social support and self-medication with alcohol or substances. Multiple and longer previous hospitalizations linking with higher risk for relapses also confirm findings that admissions for BPD should be brief and focused on managing acute risk. The therapeutic strategy should be followed by structured psychotherapy to lessen the risk of reinforcing dysfunctional help-seeking behaviors and dependence on emergency care. All these factors should permit the patients to improve their quality of life and functioning. Discharge plans, psychoeducation groups, and the availability of a stepped crisis support model, such as brief admissions, could be other effective interventions to help reduce the burden on inpatient services.

Caregiver burden evaluation and support could also be a promising direction for research going forward, building on data recognizing the importance of the patient’s home environment and social integration in promoting the recovery. Analysis of a wider, multicentric sample and the inclusion of pharmacotherapy at discharge and rehospitalization, as well as other therapeutic strategies activated in the outpatient setting, could help to better characterize patient subpopulations and deepen understanding into which care options could be better suited to which patient subcategory.

## Data Availability

The data presented in this study are available on request from the corresponding author. The data are not publicly available due to privacy/ethical restrictions.

## Long descriptions

### Table 1. Long description

The table consists of six columns: Characteristics, Total sample (N = 117), Rehospitalized (N = 55), Non-rehospitalized (N = 62), t/chi-squared value, and p-value.

* Gender (female): Total 84 (71.8); Rehospitalized 37 (67.3); Non-rehospitalized 47 (75.8); t/chi-squared 1.048; p .306.

* Current age in years: Total 33.56 plus or minus 13.73; Rehospitalized 34.60 plus or minus 13.55; Non-rehospitalized 32.65 plus or minus 13.94; t/chi-squared minus .767; p .445.

* Marital status (Overall chi-squared 8.045; p .045):

- Single: Total 53 (45.3); Rehospitalized 22 (40.0); Non-rehospitalized 31 (50.0).

- Married: Total 25 (21.4); Rehospitalized 8 (14.5); Non-rehospitalized 17 (27.4).

- Divorced: Total 33 (28.2); Rehospitalized 22 (40.0); Non-rehospitalized 11 (17.7).

- Widowed: Total 6 (5.1); Rehospitalized 3 (5.5); Non-rehospitalized 3 (4.8).

* Educational level in years: Total 11.80 plus or minus 2.95; Rehospitalized 11.45 plus or minus 2.99; Non-rehospitalized 12.11 plus or minus 2.90; t/chi-squared 1.209; p .229.

Navigate back to Table 1..

### Table 2. Long description

The table consists of six columns: Clinical Characteristic, Total Sample (N = 117), Rehospitalized (N = 55), Non-rehospitalized (N = 62), t/chi-squared value, and p-value. Significant findings (p < .05) include:

* Duration of hospitalization in days: Total 10.51 plus or minus 7.73; Rehospitalized 12.18 plus or minus 7.83; Non-rehospitalized 9.03 plus or minus 7.39 (p = .027).

* Number of hospitalizations: Rehospitalized patients had a higher frequency of 5 or more hospitalizations (47.3 percent) compared to non-rehospitalized (24.2 percent) (p = .027).

* Lifetime suicide attempts: High-lethality attempts were more frequent in the rehospitalized group (32.7 percent) than the non-rehospitalized group (12.9 percent) (p = .036).

* Number of suicide attempts: Rehospitalized 2.05 plus or minus 2.67; Non-rehospitalized 1.06 plus or minus 1.72 (p = .017).

* Current alcohol abuse: Rehospitalized 56.4 percent; Non-rehospitalized 33.9 percent (p = .015).

* Current substance abuse: Rehospitalized 60.0 percent; Non-rehospitalized 32.3 percent (p = .003).

Non-significant characteristics include family history for psychiatric disorder, involuntary admissions, current suicide ideation, current suicide attempt lethality, and medication abuse.

Navigate back to Table 2..

### Table 3. Long description

The table consists of six columns: Clinical Dimension, Total sample (N = 117), Rehospitalized (N = 55), Non-rehospitalized (N = 62), t/chi-squared, and p-value. Significant p-values (p < .05) are noted for several dimensions.

* A A S P (Adolescent/Adult Sensory Profile) scores show no significant differences across Low registration, Sensation seeking, Sensory sensitivity, or Sensation avoiding.

* Beck hopelessness scale: Rehospitalized (11.80 plus or minus 5.75) vs Non-rehospitalized (9.63 plus or minus 5.68), p = .042.

* Toronto alexithymia scale–20 (T A S–20) Total: Rehospitalized (60.05 plus or minus 14.70) vs Non-rehospitalized (52.29 plus or minus 15.29), p = .006.

* T A S–20 subscales: D D F (p = .028) and E O T (p = .001) are significant; D I F (p = .104) is not.

* C O P E problem-focused coping: Instrumental social support is significant (p = .015), with higher scores in the non-rehospitalized group.

* C O P E emotion-focused coping: Social-emotional support (p = .001) and Positive reinterpretation and growth (p = .006) are significantly higher in the non-rehospitalized group.

* C O P E potentially disadaptive strategies: Alcohol and drug disengagement is significantly higher in the rehospitalized group (10.02 plus or minus 3.20) compared to non-rehospitalized (8.23 plus or minus 2.91), p = .002.

Navigate back to Table 3..

### Table 4. Long description

The table consists of four columns: Predictor Variable, p, H R (Hazard Ratio), and 95 percent C I (Confidence Interval).

* Marital status (divorced): p equals .014, H R equals 2.025, 95 percent C I is 1.154 to 3.554.

* Current substance abuse: p equals .031, H R equals 1.868, 95 percent C I is 1.059 to 3.296.

* T A S-20_E O T: p equals .035, H R equals 1.063, 95 percent C I is 1.004 to 1.126.

* C O P E_Use of social-emotional support: p equals .034, H R equals .887, 95 percent C I is .793 to .991.

* C O P E_Alcohol and drug disengagement: p equals .026, H R equals 1.103, 95 percent C I is 1.012 to 1.204.

Navigate back to Table 4..

### Figure 1. Long description

A forest plot with a horizontal x-axis ranging from 0 to 4. A vertical reference line is positioned at 1. Five variables are listed on the y-axis from top to bottom.

* C O P E underscore Use of social-emotional support: The data point is approximately 0.75, located to the left of the reference line with a narrow confidence interval.

* T A S-20 underscore E O T: The data point is approximately 1.1, slightly to the right of the reference line with a very narrow confidence interval.

* C O P E underscore Alcohol and drug disengagement: The data point is approximately 1.15, slightly to the right of the reference line with a narrow confidence interval.

* Current substance abuse: The data point is approximately 1.8, with a wide confidence interval extending from 1.1 to 3.2.

* Marital status divorced: The data point is approximately 2.1, with a wide confidence interval extending from 1.15 to 3.5.

Navigate back to Figure 1..

### Figure 2. Long description

The x-axis is labeled Time to re-hospitalization in months with increments from 0 to 12. The y-axis is labeled Cumulative Survival with decimal increments from 0,0 to 1,0. The data is represented by a blue step-down line. At month 0, survival is at 1,0. A sharp drop occurs at month 1 to approximately 0,88. The line continues to step down at each monthly interval. By month 6, the cumulative survival is approximately 0,62. Between months 7 and 9, the rate of decline slows, plateauing near 0,6. A final drop occurs between months 10 and 11, ending at month 12 with a cumulative survival of approximately 0,55, marked by a small blue plus symbol.

Navigate back to Figure 2..
